# Barriers, facilitators, and recommendations for sexual orientation and gender identity data collection in community oncology practices

**DOI:** 10.1002/cam4.6517

**Published:** 2023-09-21

**Authors:** Megan A. Mullins, Lisa Reber, Ariel Washington, Marina Stasenko, Aaron Rankin, Christopher R. Friese, Mary E. Cooley, Matthew F. Hudson, Lauren P. Wallner

**Affiliations:** ^1^ Peter O'Donnell Jr. School of Public Health UT Southwestern Medical Center Dallas Texas USA; ^2^ Harold C. Simmons Comprehensive Cancer Center UT Southwestern Medical Center Dallas Texas USA; ^3^ Karmanos Cancer Institute, Department of Oncology Wayne State University School of Medicine Detroit Michigan USA; ^4^ Division of Gynecologic Oncology, Department of Obstetrics and Gynecology NYU Langone Health New York New York USA; ^5^ Department of Internal Medicine University of Michigan Ann Arbor Michigan USA; ^6^ Center for Improving Patient and Population Health University of Michigan Ann Arbor Michigan USA; ^7^ Rogel Cancer Center University of Michigan Ann Arbor Michigan USA; ^8^ Phyllis F. Cantor Center, Research in Nursing and Patient Care Services Dana‐Farber Cancer Institute Boston Massachusetts USA; ^9^ Prisma Health Cancer Institute Greenville South Carolina Greenville South Carolina USA

**Keywords:** clinical cancer research, clinical guidelines, epidemiology, psychosocial studies

## Abstract

**Background:**

Sexual orientation and gender identity (SOGI) data collection in community oncology practices is critical to identify and address cancer inequities, but less than 20% of NCI Community Oncology Research Program (NCORP)‐affiliated practices regularly collect SOGI data despite widespread recommendations. We evaluated multilevel barriers and facilitators for SOGI data collection at NCORP practices.

**Methods:**

We conducted 14 semi‐structured interviews at seven purposefully sampled NCORP oncology practices. We interviewed one clinician (oncologist, advanced practice provider) and one clinic staff member per practice. Thematic analysis informed by the Consolidated Framework for Implementation Research (CFIR) was conducted to identify barriers and facilitators.

**Results:**

Thematic saturation occurred after interviews at six practices and was confirmed with interviews at an additional practice. Participants highlighted multilevel barriers including low levels of understanding, information technology infrastructure, and perceived low relative priority. Not understanding the role of SOGI data in oncology care contributed to cis‐heteronormative culture. At the clinic level, this culture coincided with a lack of processes and policies for collecting SOGI from all patients. At the care team level, perceived irrelevance to oncology care was related to discomfort asking SOGI, fear of patient discomfort, and limited awareness of SOGI in electronic health records. Suggested solutions included: normalizing asking SOGI questions, giving patients privacy to complete SOGI, and clarifying clinical relevance.

**Conclusions:**

SOGI data collection barriers stemmed from perceptions that SOGI disclosure does not influence care quality. Oncology teams may benefit from training on culturally sensitive SOGI collection, education on SOGI data relevance to oncology practices, and support for implementing SOGI data collection policies.

## 1 INTRODUCTION

In addition to higher cancer risk^1^, ^2^, ^3^, ^4^ and later stage cancer diagnoses,^5^ sexual and gender minority (SGM) individuals experience lower satisfaction with their cancer care, more distress during survivorship, care delays, and unmet patient‐provider communication needs.^6^, ^7^, ^8^, ^9^, ^10^ The Joint Comission,^11^ National Academies of Medicine,^12^ Department of Health and Human Services^13^ and American Society for Clinical Oncology (ASCO),^14^ all provide guidance on sexual orientation and gender identity (SOGI) data collection, as these data are essential to identify, understand, and inform solutions for disparities throughout the cancer care continuum. Systematic collection of SOGI data is essential to prioritize cancer control interventions in this population. However, practitioners do not routinely collect SOGI data in electronic health records (EHRs), and reasons why they do not routinely and systematically collect these data are unclear.^15^, ^16^, ^17^, ^18^, ^19^ In a 2020 ASCO member survey, respondents identified leadership support, dedicated resources, and individual respondent attitudes as facilitators for SOGI data collection. Most of these respondents were clinicians practicing at academic medical centers, thus limiting the generalizability to community oncology settings.^19^

Community oncology practices deliver the majority of cancer care, but we have limited information regarding SOGI data collection in these practices.^20^ To bring research advances to practices throughout the United States and improve generalizability of study findings, the NCI Community Oncology Research Program (NCORP) includes 46 community sites accruing people with cancer to cancer clinical trials, research, and care delivery studies.^20^ A 2017 assessment found that 72% of NCORP practice groups reported they did not collect sexual orientation nor gender identity information.^21^ However, studies indicate patients are willing to disclose SOGI and often do so.^22^, ^23^ Practices in the western United States and practices in states with higher proportions of SGM individuals in their state were more likely to collect SOGI, though researchers did not clarify practice‐level barriers and facilitators of SOGI data collection.^21^ Barriers and facilitators to SOGI data collection in community oncology practices are likely multilevel (patient‐, provider‐, clinic‐). At the patient level, practice setting, patient/provider rapport, and provider specialty contribute to patient comfort with disclosing SOGI information, but there is a dearth of information regarding provider and clinic‐level barriers and facilitators to SOGI data collection in community practice.^22^, ^23^ Therefore, we conducted a qualitative study of physicians, advanced practice providers, and clinical staff that included semi‐structured interviews to understand provider‐ and clinic‐level barriers and facilitators to SOGI data collection, and recommend strategies to enhance SOGI data collection.

## 2 METHODS

Investigators used the Consolidated Criteria for Reporting Qualitative Research (CORE‐Q) checklist to guide reporting.^24^

### 2.1 Study design and recruitment

Content analysis was the methodologic orientation for this study.^25^ Investigators purposively sampled NRG Oncology member NCORP sites via email. NRG Oncology's community practice affiliates are a geographically diverse sample, as indicated by region, rural/urban commuting area, and patient mix (minority underserved status vs. not designated as minority/underserved). We emailed an invitation for practice participation to NCORP research administrators at NRG Oncology member sites with an active site PI (32 of 40 national NCORP sites). Administrators at interested sites identified practices with a physician or advanced practice provider and a staff member at their practice to participate in a 30‐min virtual interview. To ensure practice participation representing more diverse patient representation, we specifically targeted two minority underserved sites for practice participation with direct emails to a site physician. The University of Michigan Institutional Review Board deemed this study exempt under exemption category three criteria A and B, and did not require written consent. However, investigators obtained verbal consent prior to each interview. Participants received a $40 electronic Amazon gift card upon interview completion.

### 2.2 Data collection

Interviews were conducted from February 21, 2022 to June 3, 2022. Data included participants' professional role and years of experience in their current position, as well as practice location information from their institution's webpage. A cancer care delivery researcher experienced in qualitative research (MAM) conducted and recorded all interviews virtually (via Zoom platform) using a semi structured interview focused on eliciting SOGI data collection processes and perceived barriers and facilitators. Participants joined virtually from an isolated location at their workplace or home. Participants had no prior relationship with the interviewer. The Consolidated Framework for Implementation Research (CFIR) and a priori knowledge informed interview guide development (Data S1).^26^ Interviews lasted between 15 and 30 min and were only conducted once with each participant. Thematic saturation was achieved after 12 interviews, and investigators solicited interviews at one additional site to confirm themes.

### 2.3 Analysis

Interview transcripts were automatically generated via the Zoom platform. Students reviewed and cleaned the transcripts with the recordings, and LR did a final check and ensured verbatim transcription. The study team used thematic analysis to evaluate barriers and facilitators to SOGI data collection in oncology clinics.^27^ Interview transcripts were reviewed and coded by three independent coders (MAM, LR, AR) using NVivo Release 1.7 (QSR International). An initial codebook was generated a priori based on constructs included in the updated CFIR framework.^28^ ^(p2)^ Initial coding was conducted deductively using CFIR as a starting point. Additional codes and themes were identified inductively to address those aspects of participant narrative not captured by the existing CFIR constructs, and descriptions of codes were adjusted to better reflect the patterns identified. Codes were reviewed using a constant comparative method and discrepancies were resolved by discussion and consensus. Saturation was discussed as interviews were coded and reviewed. After coding, two coders (MAM, LR, a PhD trained qualitative methodologist) reviewed coded excerpts independently and met to discuss and clarify emerging themes. Key themes and exemplary quotes were selected.

## 3 RESULTS

Seven NCORP practices from five geographic regions in the US participated, including three minority/underserved NCORP sites (Table 1). One interested NCORP site was unable to find two interview participants, and one clinician who was contacted for interview did not respond, requiring the site administrator to identify another clinician from that site. All other individuals contacted consented to interview and participated. Clinicians (two medical oncologists, two oncology advanced practice providers, and three gynecologic oncologists) reported an average of 4.9 years at their current practice and staff reported an average of 4.8 years.

**TABLE 1:** Practice and Participant characteristics.

Practice number	US region	Rural	Minority underserved	Clinician (years at practice)	Staff (years at practice)
1	West	No	Yes	Medical oncologist (3.5)	Patient services representative (5)
2	Midwest	No	No	Oncology APP (3)	Medical assistant (6)
3	Upper Midwest	No	No	Oncology APP (7)	Social worker (4)
4	Midwest	Yes	No	Oncologist (6)	Medical assistant (5)
5	Midwest	No	No	Gynecologic oncologist (8)	Front desk staff (5.5)
6	Northeast	No	Yes	Gynecologic oncologist (4)	Medical assistant (3)
7	South	No	Yes	Gynecologic oncologist (3)	Clinic assistant/surgery scheduler (N/A)

Abbreviation: APP: advanced practice provider (nurse practitioner or physician assistant).

**FIGURE 1:**
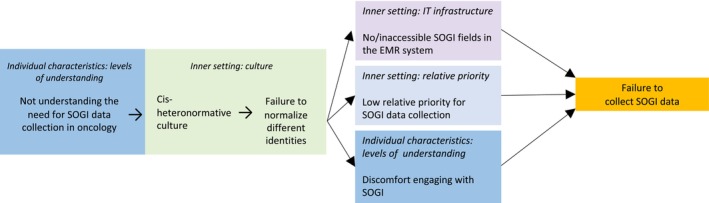
Model of oncology clinician and staff reported SOGI data collection barriers and facilitators.

Figure 1 presents identified barriers and facilitators to SOGI data collection. These factors fell predominantly into the individual characteristics and inner setting CFIR domains. Within the individual characteristics domain, we identified an additional construct called “levels of understanding”, composed of two broad themes: (1) understanding the need for SOGI data collection and (2) comfort engaging with SOGI. In the inner setting domain, broad themes aligned with the existing CFIR constructs (3) culture and (4) relative priority. Table 2 presents themes, sub‐themes, and exemplary quotes.

**TABLE 2:** Oncology clinician and staff reported barriers and facilitators to sexual orientation and gender identity data collection.

CFIR domain: construct	Theme/barrier	Exemplary quotation	
		Staff	Clinicians
Individual characteristics: Levels of understanding^a^	Understanding need for SOGI collection	*[Understanding relationship between SOGI collection and patient care]* “I feel like it [collecting SOGI] would be an important part of collecting data, because you don't want anybody to feel, I don't know, like they're not in the system correctly.” (Medical Assistant, H) *[Not understanding relationship between SOGI collection and patient care]* “I feel like it doesn't, in my field at the cancer center, it [SOGI] doesn't matter what you have, you have cancer, we're not here to treat you for something related to your identity.” “we're here for the cancer, not learning that stuff.” (Medical Assistant, D) *[Wanting to understand reason for SOGI collection]* “I mean I wish I did know [why we asked about SOGI]. [Laughter] If I had that information, then it would be totally fine.” (Medical Assistant, L)	*[Understanding relationship between SOGI collection and patient care]* “I would say just an awareness of how that plays into a person's health care is something that is, that awareness, on the provider, and even staff level, it's just, there's a gap there.” (APP, C) *[Not understanding relationship between SOGI collection and patient care]* “No [there are no barriers at my practice to being able to collect SO], my only thought would be, is why we would ask … it really doesn't play a role in caring for our patient.” (Oncologist, G) *[Wanting evidence supporting need for SOGI collection]* “I guess if there was data that showed that we had better outcomes or patients were more comfortable.” (Oncologist, I)
	Comfort engaging with SOGI	*[Comfort with SOGI collection]* “Here at the facility that I work for, we embrace the LGBTQ community, and we make it safe for them to voice, safe for them to feel seen, safe for them to come and seek treatment.” (Scheduler, M) *[Low self‐efficacy]* “[I'm] not really comfortable asking [patients about SOGI or responding to questions why I'm asking] because I wouldn't have a response for that, so that's what makes me a little uncomfortable.” (Medical assistant, L) *[Assumptions about patients' reaction to SOGI]* “[Patients] feel too awkward to put down what they are, … what they identify.” (Medical Assistant, D) *[Why staff are reluctant to engage on topic of SOGI]* “I think people have concerns about offending somebody or getting it wrong or some of that, so there probably is some avoidance in regards to that…. There's like a polite kind of culture up here as well that I think becomes a barrier and people don't want to like create problems, but, often that stifles a lot of communication.” (Social Worker, F)	*[Low self‐efficacy]* “From a provider … standpoint, I guess the only barrier … that I can think of, is just not being comfortable asking the questions, or not being fully educated or informed about sexual orientation, … I think it's an area, personally, that I need more education on, something I just need to get more comfortable with in order to incorporate it into practice.” (APP, E) *[Assumptions about others' reactions to SOGI]* “[Staff might be] uncomfortable asking [… and patients might] not appreciate.” (Oncologist, B) *[Why staff are reluctant to engage on topic of SOGI]* “So I think a lot of times providers or medical assistants, or nurses feel just not used to. It's like a muscle they're not used to flexing: asking patients what their gender identity is, or even starting the conversation.” (APP, C) “Yeah, I mean everyone messed up all the time. I mess up all the time. But I think the thing about it is all that matters to this [transgender] patient is that people care about their feelings and are trying.” (Oncologist, K)
Inner setting: Culture	Culture of cis‐heteronormativity	*[Normalizing non‐normative identities]* “We do modules like they keep us abreast, of what's going on in the community and outside and abroad, and we do modules on how to be caring, compassionate, and how to address them, how to use proper pronouns, how to make them feel welcome, how to make them feel heard.” (Scheduler, M) *[Making SOGI questions routine]* “We've encouraged [staff] to ask in the visits, during initial visits, as well as ongoing and just kind of normalizing it as a professional behavior that we would do in any setting.” (Social Worker, F) *[Assuming Cisgender]* “Well, we're in a gynecology office, obviously, so everybody's marked female unless they let us know otherwise.” (Front desk, J) *[Selectively supporting queer or trans‐patients when need is detected]* “If I get any hint or observation that maybe somebody is on some sort of spectrum, or has marginalized identity, I will engage in that and ask more questions and do more assessment. But I would say, if I'm being honest, that I am inconsistent.” (Social Worker, F)	*[Normalizing non‐normative identities]* “We are trying to improve our gender and sexual inclusivity here at our facility as a broad organization. …. They're just trying to make sure that we reduce …. ah, stigma as much as possible.” (APP, C) *[Making SOGI questions routine]* “We're trying to figure out, what can we do to support this and make it as seamless as possible, so that we can start just making this a regular like race‐ethnicity type of questions.” (Oncologist, G) *[Assuming heterosexual]* “It becomes uncomfortable for me when maybe it was something I didn't know ahead of time. Um, like, um, maybe, you know if that patient offers up something, and I think oh, my goodness, I didn't know that, I should have known that.” (Oncologist, K) *[Placing onus of disclosure on patient]* “Where practices see mostly cis women …I don't know how much presumption there is. I worry that there might be more than there ought to be. Patients might need to speak up, you know, in order to get their preferred gender.” (Oncologist, K) *[Lacking knowledge]* “I believe [there are questions about sexual orientation]. Is sexual orientation whether they're heterosexual, not gender identity?” (Oncologist, N)
Inner setting: Relative priority	Priority for SOGI data collection	*[High priority in organization]* “Our organization has really stepped forward … health care equality being essential across the board. I think we take pride in that … We do have a department specifically that handles equalities, cultural, race, sexual‐identification expression.” (Registration, A) *[High priority to individual]* “I am personally super out for it and I think that it's important.” (Social worker, F)	*[High priority in organization]* “Our institution has instituted a standard way that that is collected. I think that's probably why it's just more readily seen … more receptive to it.” (Oncologist, I) *[High priority to individual]* “I think as part of the intake process, it should be part of that.” (Oncologist, E) *[Lesser priority to colleagues]* “I don't think that the attitude is against the question. I think the attitude is against more work … a load of work issue.” (Oncologist, G) *[Less priority to individual]* “I do not really recall that. [Questions about sexual orientation] may be on there. … I'm going to check my phone … It's funny you say that… because I do not know.” (Oncologist, N)

Abbreviations: APP, advanced practice provider; SOGI, sexual orientation and gender identity.

^a^ This is a construct not addressed by CFIR.

### 3.1 Individual characteristics: levels of understanding

#### 3.1.1 Understanding the need for SOGI collection

Respondents commonly lacked understanding of the need for SOGI data collection in oncology clinics but expressed an interest in learning more (Table 2). Many indicated that knowing patient gender identity was more important than knowing their sexual orientation because gender identity influences how to respectfully address patients. In contrast, all physicians and almost all staff indicated patient's sexual orientation was not essential to their work and could not understand its relevance: “I have to admit that I don't routinely address [sexual orientation] during my visits, because usually I'm seeing, I'm kind of focused on the cancer issue” (Oncologist, I). Staff and clinicians were often unaware of how recognition of an individual's sexual orientation may impact their care. When physicians did acknowledge the importance of sexual orientation, they linked SOGI's import to patient social support. Respondents also indicated that staff made their own determination of patients' SOGI: “If patients don't answer, or they don't get asked it, then it kind of falls unfortunately on the person inputting the information to kind of make it up… Staff I think are guessing in instances.” (Social Worker, F).

#### 3.1.2 Comfort engaging with SOGI

In general, when asked about their comfort with SOGI data collection, participants' responses were framed around understanding, acceptance, and inclusivity (Table 2). We also found participant comfort to be tied to their sense of self‐efficacy. Those who lacked self‐efficacy indicated anxiety and fear of offending, creating discomfort, or encountering conflict. For example, a woman who worked at the front desk said it was typically older patients who would become confrontational and tell her, “This isn't any of your business, why are you asking me this?” Both staff and physicians described the discomfort they experienced because they lacked the knowledge and language they believed they needed to engage on the topic of SOGI effectively. A medical assistant reported discomfort when she could not “explain why the question was being asked.” Interestingly, although some participants were frank in disclosing their discomfort, many instead spoke about why their colleagues might be reluctant to engage with patients on the topic or why patients might hesitate to respond or express anger upon at being asked.

### 3.2 Inner setting

#### 3.2.1 Cis‐heteronormative culture

Within the inner setting, participants highlighted cultural assumptions of patients being cis‐gender and heterosexual. For example, assuming that all patients were cisgender women in a gynecological oncology setting. These assumptions necessitated dedicated efforts from patients and staff to ensure transgender patients were respected and provided appropriate care. One oncologist reported an episode when staff were calling for the next patient but kept overlooking the appropriate patient because the care team presumed a female gender, when in fact the patient was a transgender man. This theme also captures the lack of consistency and selectivity produced when providers rely exclusively on appearance (e.g., “dressing masculine”) to identify patients' sexual and gender identities, thereby selectively targeting individuals for disclosure and otherwise placing upon the patient the onus of correcting providers' misconceptions of gender and sexual orientation (Table 2).

#### 3.2.2 Low relative priority for SOGI data collection

Relative priority of SOGI data collection was one of the central themes we identified, and it varied by job function. Staff often considered SOGI data importance relative to organizational concerns: “We do have like a whole department that comes out and does in‐services and brings awareness; they also send out various emails. I think next week we're having a LGBTQ parade that all are invited to in the hospital.” (Scheduler, M). Clinicians considered SOGI data collection challenges relative to care impact and capacity. One physician explained workload influenced physicians' perspectives:

In order to understand the questions that you're asking, I think you need to get a real understanding of the burden that charting is to clinicians because I think what you're saying is like, “look, but it's important for doctors to know this thing about patients that is extremely important to patients’ lives, and therefore to chart it,” right, and I think that doctors would say to you, It's like “don't you dare make me press another button.” (Oncologist, K)

This physician appeared to appreciate the potential importance of SOGI collection. However, another physician observed, with “the hustle and bustle” SOGI data collection was not “the most critical thing…maybe it gets left on the side.” (Oncologist, B).

#### 3.2.3 IT Infrastructure

Results indicate variation in EHR formats for SOGI data collection. Ascertaining whether practices had discrete fields for SOGI data collection in their EHR was challenging due to contradictory responses from participants at the same practice about the presence and location of SOGI fields, participants confusing SO and GI terms, and low awareness of SOGI data fields. Participants also emphasized patient‐level barriers and opportunities to better utilize patient portals for SOGI data collection. Table 3 provides examples of these IT infrastructure barriers and facilitators.

**TABLE 3:** Electronic health record related barriers and facilitators for sexual orientation and gender identity data collection.

CFIR domain: construct	Theme/barrier	Example quotation
Inner setting: IT infrastructure	Awareness of SOGI data fields	*[Increase awareness of SO fields]* “I'm not 100% sure I could find it.” (Oncologist, B) [Awareness of SO fields location] “Where do they have [sexual orientation]? … In the demographics. I'm just trying to see if we have it on the … I know it's on… one of the systems that we use … hmmm. That's odd. You never can locate anything when you need it.” (Scheduler, M) [Workaround for no SOGI fields] “When I go to enter in the vitals, there's actually a section that says “edit tobacco and drink usage”, and at the very bottom of that is where [staff] plug it in at.” (Medical assistant, D)
	Ease of access to SOGI fields	[Ease of access to SO fields] “Lesbian or gay, it's not easily accessible.” (Oncologist, I) [Ease of access to GI fields] “Very nice … on that left‐hand side … don't need to expand it” (Oncologist, C) [Ease of access to GI fields] “You would have to search for it, because now I'm looking in here and I do see there is a section for sexual orientation and gender identity, but I didn't know about it; I didn't know we had it in there, actually. … It's just not easy to find.” (Medical Assistant, H)
Inner setting: Patient process	Awareness of SOGI fields	“I know there is a section for [sexual orientation and gender identity] in MiChart but I don't think a lot of [patients] know how to get to it.” (Medical assistant, D)
	Ability to change SOGI information	“I do not know of them being able to change anything as far as their demographics on the online portal, it's just a view, the portal that we have” (Registration, A)
	Preference for entering SOGI information	“[Sexual orientation is a question] but a lot of them … they just put it … they don't even fill it up…. The only thing that I can say that they will fill out is preferred language.” (Medical assistant, L)

Abbreviations: GI, gender identity; SO, sexual orientation; SOGI, sexual orientation and gender identity.

#### 3.2.4 Key recommendations

Table 4 summarizes staff and clinician recommendations for SOGI data collection. In addition to EHR infrastructure changes, respondents suggest collecting SOGI data in clinical portals or paper forms to enhance SOGI data utility in clinical settings. Several also mentioned normalizing SOGI data collection for all patients so that staff and clinicians can use “a muscle they are not used to flexing.”

**TABLE 4:** Oncology clinician and staff recommendations for sexual orientation and gender identity data collection.

Recommendation	Theme/barrier addressed
[Training] Provide explanations of why staff and clinicians should be knowledgeable about SOGI (e.g., how it impacts care/outcomes, evidence of patient preferences) [Training] Use patients, speakers from the LGBQ community, and trusted experts to share patient stories. [Training] Provide script/dialog to staff for asking NASEM SOGI questions and for responding and engaging with patient questions [Intake process] Ask all patients their SOGI to normalize the questions in demographics collection	Understanding need for SOGI collection Comfort engaging with SOGI Normalizing non‐normative identities Making SOGI a priority
[IT infrastructure] Include discrete SOGI fields [IT infrastructure] Position SOGI fields with other demographic data to ease data input [IT infrastructure] Display SOGI data as part of storyboard/banner to facilitate clinician awareness and access	Staff awareness of SOGI data fields Clinician ease of access to SOGI fields
[IT infrastructure] Include discrete SOGI fields for data entry and editing in patient portal [IT infrastructure] Position SOGI fields with other demographic data to ease data input [IT infrastructure] Allow patients to disclose SOGI in patient portals or questionnaires prior to their visit	Patient awareness of SOGI fields Patient ability to change SOGI information Patient preference for entering SOGI information

Abbreviations: IT: information technology; LGBQ, lesbian, gay, bisexual, and queer; NASEM, National Academies of Science, Engineering, and Medicine; SOGI, sexual orientation and gender identity.

## 4 DISCUSSION

In this national sample of community oncology practices, we identified patient‐, staff‐, clinician‐, and clinic‐level barriers to SOGI data collection. Respondents reported similar barriers across sites that included suboptimal understanding of SOGI data collection need, cis‐heteronormative culture in clinical settings, missing or buried SOGI data fields in the EHR, competing patient data collection priorities, and discomfort soliciting SOGI due to fear of producing potential patient discomfort or upset. Most cancer care is delivered in community oncology settings, yet to our knowledge, ours is the first study to provide in‐depth evaluation of barriers and facilitators to SOGI data collection in community oncology practices in the United States.

Our study builds on prior work by providing important contextual information from community oncology settings. The 2017 NCORP Landmark Survey found that only one in five NCORP practice groups regularly collected SOGI data.^21^ Investigators found NCORP practices with more ethnic diversity and higher proportions of SGM patients were more likely to collect SOGI data. However, these relationships remain poorly understood.^21^ A 2020 ASCO survey found institutional support, dedicated resources, and individual respondent attitudes were necessary for SOGI data collection.^19^ In open ended responses they also identified institutional culture, provider beliefs and discomfort, patient discomfort, lack of EHR fields, and lack of training, resources and time as important barriers.^19^ These results reflect clinician views from predominantly academic medical centers, which may explain the slight differences we observed in community practice settings. Like the ASCO study, we found that culture influenced SOGI data collection, but participants in our study often spoke about leadership and culture on a clinic level rather than an institutional level. Although some participants mentioned hospital system Diversity, Equity and Inclusion initiatives supported SOGI data collection, clinicians described more granular leadership support for SOGI data collection that included EHR setup and asking their clinic staff to “click a button” and populate valued information as necessary leadership for SOGI data collection. Clinical staff also referenced the welcoming environment their clinical team provides, suggesting that change initiatives and education should target clinic level leadership, not only institutional leadership, in community oncology settings.

Similar to other studies, many participants did not perceive SOGI to be important for providing high quality care, especially physicians.^29^, ^30^ Understandably, we found that ignorance about the benefit of SOGI data collection in oncology deprioritized SOGI data collection relative to other clinical topics in short oncology visits and led to discomfort with SOGI. While most responses about comfort with SOGI were personal, descriptions of discomfort were commonly displaced so that the respondent was reflecting on how they thought their patients or colleagues might feel. Considering that the 2020 ASCO survey also found more than 30% of respondents believed that their patients would be uncomfortable if asked about their SOGI, widespread discomfort is an important misconception that must be corrected in trainings.^19^ A growing body of literature demonstrates not only patient willingness to disclose SOGI, but improved self‐reported health among SGM individuals when they can disclose.^31^, ^32^, ^33^ Addressing underlying discomfort is critical because engaging with SOGI creates vulnerability for patients, clinicians, and staff. This can be addressed with training to improve self‐efficacy in collecting and engaging with SOGI data, and creating an ethos of cultural humility that includes self‐reflection on biases and curiosity about others' identities.^34^ Part of cultural humility is acknowledging that mistakes can happen, taking the correction, and continuing to learn. As one gynecologic oncologist explained, “It's not that everyone always gets the pronouns right every time It's that people don't just keep misgendering them over and over again and seeming to do it in a way that's disregarding of their preferences.” We can nurture a culture of curiosity and respect to assuage care team discomfort negotiating unfamiliar terms. Though everyone can benefit from cultural humility in a clinical setting, it is likely critical to offer different trainings, training delivery, and training incentives for physicians and staff. Staff were more willing to admit their lack of knowledge about SOGI and express an interest in learning. Providers framed their need for more knowledge around the absence of data showing better outcomes. They also spoke about time issues and other commitments, while the staff did not.

This study has potential limitations that warrant comment. First, we sampled clinicians and staff who have access to clinical and cancer care delivery trials through the NCORP. These practices likely have different practice environments and patient populations than community oncology practices that do not participate in trials. However, we did purposively sample a national group of practices to maximize diversity in geographic region, rurality, and minority underserved status. We have the added strength of recruiting through the NCORP research administrators at each practice who connected us with participants, so interviewees did not self‐select into the study due to personal interests in SOGI. Second, our sample size was small. However, we did confirm thematic saturation, and previous research suggests coding becomes relatively stable after 12 interviews, in some cases even fewer.^35^, ^36^ Third, given our recruitment strategy, participation was based on the availability and interest of site contacts and we cannot measure refusal to participate. Though we took steps to maximize diversity such as including practices from five US regions and three minority underserved sites (which reflect racial/ethnic or rural patients), it is possible there could be selection bias in our sample. Finally, in many cases, we could not discern whether practices were regularly collecting SOGI data due to contradictory responses between participants, lack of awareness of SOGI, and conflation of SOGI. While greater insight would have been informative, participants' difficulty in articulating the difference is indicative of the training that is needed, even in practices where prior training has occurred.

Nearly 10% of the US population identified as SGM in a 2021 national survey, a number that is higher among young adults and rising.^37^ Thus, we must acknowledge the importance of SOGI data collection in oncology care. Studies show SGM individuals experience lower satisfaction with their cancer care, more distress during survivorship, delays in care, and unmet needs in patient‐provider communication.^6^, ^7^, ^8^, ^9^, ^10^ Not only does SOGI data inform equitable, patient‐centered care, tailored resources, and reduced minority stress, SOGI disclosure itself may help reduce delays in care seeking.^38^ Based on our findings, training clinicians and staff, and EHR changes are essential strategies to support SOGI data collection. Providing discrete fields in the EHR, positioning those fields in ways that flow with patient intake/registration, and asking all patients SOGI questions may normalize the process and encourage sustained‐data collection efforts. However, respondents also encouraged opportunities outside of clinic such as forms or patient portals. The utility of portals or non‐verbal modes of collection is echoed in patient studies eliciting preferences for SOGI disclosure.^32^, ^39^

## 5 CONCLUSIONS

In this study of NCORP practices, we identified several barriers to SOGI data collection stemming from perceptions that SOGI disclosure does not influence cancer care quality. In addition to clarifying how SOGI data collection can improve patient experiences and inform care, efforts should include addressing underlying discomfort around engaging with SOGI. Oncology teams may benefit from training on culturally sensitive SOGI collection, education on SOGI data relevance to oncology practices, and support for implementing SOGI data collection policies.

## AUTHOR CONTRIBUTIONS

**Megan A. Mullins:** Conceptualization (lead); data curation (lead); formal analysis (lead); funding acquisition (lead); investigation (lead); methodology (lead); project administration (lead); resources (lead); supervision (lead); writing – original draft (lead). **Lisa Reber:** Formal analysis (equal); writing – original draft (equal); writing – review and editing (equal). **Ariel Washington:** Formal analysis (equal); writing – review and editing (equal). **Marina Stasenko:** Conceptualization (equal); data curation (equal); writing – original draft (equal); writing – review and editing (equal). **Aaron Rankin:** Data curation (equal); formal analysis (equal); writing – review and editing (equal). **Christopher R Friese:** Conceptualization (supporting); funding acquisition (supporting); writing – original draft (equal); writing – review and editing (equal). **Mary E. Cooley:** Conceptualization (supporting); funding acquisition (supporting); investigation (supporting); methodology (supporting); writing – original draft (equal); writing – review and editing (equal). **Matthew F Hudson:** Conceptualization (supporting); funding acquisition (supporting); methodology (supporting); writing – original draft (equal); writing – review and editing (equal). **Lauren P. Wallner:** Conceptualization (supporting); data curation (equal); formal analysis (supporting); funding acquisition (supporting); investigation (supporting); methodology (supporting); supervision (supporting); writing – original draft (equal); writing – review and editing (equal).

## FUNDING INFORMATION

This work was supported by pilot funds received from NRG Oncology (NCORP grant UG1CA189867). Dr. Mullins is Supported by the Texas Health Resources Clinical Scholars Program. Drs. Friese and Mullins received research support from the National Cancer Institute institutional training grant T32‐CA‐236621. The content is solely the responsibility of the authors and does not necessarily represent the official views of Texas Health Resources, the National Institutes of Health or the National Cancer Institute. The funders had no role in the design and conduct of the study; collection, management, and analysis, and interpretation of the data; preparation, review, or approval of the manuscript; and decision to submit the manuscript for publication.

## CONFLICT OF INTEREST STATEMENT

Lauren P. Wallner: consulting role, Gilead Sciences

## Supporting information


**Data S1.** Supporting Information

## Data Availability

Data collected at the University of Michigan from participants for the IRB Exempt project titled: Sexual orientation and gender identity (SOGI) measurement for patient centered cancer care in sexual and gender minority (SGM) populations (HUM00206801). Data are being provided by the University of Michigan for this publication and research data are not shared.
