# Supplementary material for: Barriers, facilitators, and recommendations for sexual orientation and gender identity data collection in community oncology practices
Source: Cancer Med. 2023 Sep 21;12(18):19203–14. doi: 10.1002/cam4.6517 (PMC10557876; doi:10.1002/cam4.6517)
Supplement: Supplementary file 1 — Data S1. Supporting Information [file CAM4-12-19203-s001.docx]

University of Michigan

Consent To Be Part Of A Research Study

### Name of Study and Researchers

**Title of Project: Sexual orientation and gender identity measurement in community oncology practices**

**Principal Investigator: Megan Mullins, PhD, MPH**

### GENERAL Information

We’re doing a study to learn your perspective on measuring sexual orientation and gender identity in community oncology practices. To get information, we’d like 30 clinicians to participate in interviews. We expect it to take about 20-30 minutes to complete the interview.

Participating in this interview is voluntary. You don’t have to participate if you’d rather not. You can choose not to answer any questions that you don’t want to answer, whatever the reason, and you don’t have to tell us why. Choosing not to participate in the interview won’t affect your employment.

It’s possible that some of the questions may make you feel uncomfortable, though we think this is unlikely. If a question makes you uncomfortable, you can just ask to skip it and go to the next question.

To keep your information confidential, we will only keep identifiers from the audio recordings until they are transcribed, and then they will be deleted. You will not be identified in any way in any reports. Your answers will be combined with those of everyone else in the study so no one individual can be identified. We will store all records from this study on a secure server with access limited strictly to the study team. Data from this interview will not be used for future research. Data will only be used to inform the development of a clinician survey. Once the survey is developed, all audio recordings and transcriptions will be destroyed.

Participating in our interviews won’t benefit you directly. We hope what we learn will help other people in the future.

To thank you for taking part in our study, we’ll send you $40.00 after you participate in the interview. The University of Michigan accounting department may need your name, address, Social Security number, payment amount, and related information for tax reporting purposes.

### Contact Information

## To find out more about the study, to ask a question or express a concern about the study, or to talk about any problems you may have as a study subject, you may contact:

| Principal Investigator: Megan Mullins, PhD, MPH  Address: North Campus Research Complex  Bldg 16, Room 409E  2800 Plymouth Road  Ann Arbor, MI 48109  Telephone: 512-900-0349  Email: mamull@umich.edu |
| --- |

**You may also express a concern about a study by contacting the Institutional Review Board:**

University of Michigan Medical School Institutional Review Board (IRBMED)

2800 Plymouth Road

Building 520, Room 3214

Ann Arbor, MI 48109-2800
734-763-4768

E-mail: [irbmed@umich.edu](mailto:irbmed@umich.edu)

If you are concerned about a possible violation of your privacy or concerned about a study, you may contact the University of Michigan Health System Compliance Help Line at 1-866-990-0111

Aim 1 Interview Guide

Hello Dr. _____________, this is ___________. I am here

on behalf of the University of Michigan research team for a project called “Sexual orientation and gender identity measurement in community oncology practices”.

Thank you for agreeing to talk with us today. We will be speaking for 20-30 minutes.

The purpose of this interview is to learn your perspective on measuring sexual orientation and gender identity in community oncology practices. There are no right or wrong answers since people have different experiences and points of view. We value all of your insights and our team will take this information and use it to inform a study that more comprehensively examines SOGI measurement in oncology practices.

We would like to record the interview to make sure we capture everything you say accurately. Would this be OK?

**NO** – I understand. Unfortunately, for purposes of data quality, we cannot conduct any interviews if we are unable to record them. I appreciate your willingness to participate in the project. Thank you for your time and have a good day.

**YES** – During the interview, please let me know if you do not want to answer any question and I will skip to the next question.  You can also ask me to pause the recorder or stop the interview at any time. You also have the right to withdraw from the interview. This interview is purely voluntary, and your comments will be kept private. For reasons of confidentiality, we will not state your name during the interview. If we unintentionally do so, please know that it will be deleted from your interview transcript.

After the interview is finished, we will email you a $40 gift card as a small token of appreciation.

Do you have any questions for me before we begin?

I’m going to begin recording now and state some initial comments. I will then ask you for permission to record the interview so we have it recorded.

**Interviewer – turn on recording device and state:**  Today’s **date** is ____________, it is [state **time** EST], We are conducting an interview with **study ID** _______________ for the Sexual Orientation and Gender Identity measurement project.

**For our records, “Do you agree to have your interview recorded?”**

1. First, can you tell us your role and how long you have worked at your current practice?

2. What electronic medical record (EMR) does your practice use?

If no EMR, probe: can you describe how your practice collects patient information. Subsequent questions will ask about intake forms instead.

I am now going to ask you some questions about collecting information about patients’ sexual orientation. For example, lesbian, gay, straight, heterosexual, etc..

1. Does your EMR have questions about sexual orientation?

If YES:

1. Walk me through the process for populating these fields.
   1. Who populates them and when?
2. Where are they located? (example probes: demographics, risk behaviors, other?)
3. How often are these completed or filled in? PROBE: Why don’t people fill them in?

PROBES: Who fills them in, how do you access the fields, at what point of care are they completed?

1. Can patients fill in this data before their visit? Ex on an online portal

PROBE: Are you notified if they fill it in? Can you see this information easily?

If NO: how does your practice collect patient demographics like race/ethnicity?

PROBES: Do they ask on first visit when seeing them? Is there a sheet to fill out before the actual appointment? Do they not collect it at all?

1. What barriers do you think influence your practice’s ability to collect sexual orientation in the EMR? If no barriers, what is working well? What would make it easier to collect this data?

I am now going to ask you some questions about collecting information about patients’ gender identify. For example, this would include options like transgender male, and would have a question about sex assigned at birth

1. Does your EMR have questions about gender identity?

If yes:

1. Walk me through the process for populating these fields.
2. WHO fills in? where are they located? demographics, risk behaviors, other?
3. How often are these completed or filled in? PROBE: Why don’t people fill them in?

PROBES: Who fills them in, how do you access the fields, at what point of care are they completed?

1. Can patients fill in this data on an online portal before their visit?

PROBE: Are you notified if they fill it in? Can you see this information easily?

If NO:

1. how does your practice collect patient demographics like race/ethnicity?
   1. PROBES: Do they ask on first visit when seeing them? Is there a sheet to fill out before the actual appointment? Do they not collect it at all?
2. What barriers do you think limit your practice’s ability to collect gender identity in the EMR? If none, what is going well? What would make it easier to collect this data?
3. What is the process for finding out about new information/changes to your EMR?

Now I’d like to ask you some questions about your thoughts about collecting this information at your practice. **reminder that we are going to deidentify all names and positions and keep interviews confidential**

1. What is the general level of receptivity in your organization to implementing SOGI data collection?
   1. PROBE: Why?
   2. Who are individuals influential in setting the culture at your practice? [engaging- opinion leaders] Institutional culture? [inner setting- culture]
2. Can you describe for me how you feel about collecting sexual orientation and gender identity (SOGI) at your practice?

Probe: Why?

1. What type of information about the importance of SOGI data collection is needed to get staff on board?
2. Walk me through What kinds of changes you think will be needed to accommodate SOGI data collection- more often or more efficiently?
   1. PROBE 1: Changes in scope of practice? Changes in formal policies? Changes in information systems or electronic records systems? Other?
   2. PROBE 2: What kind of approvals will be needed? Who will need to be involved?
   3. PROBE 3: Can you describe the process that will be needed to make these changes?
3. Is there anything else you would like to tell me regarding SOGI data collection?

Thank you very much for your time today. We will be conducting two more sets of short (20-30 minute) interviews with Amazon cards as thanks to explore strategies for implementing SOGI measurement. Would you be willing to be re-contacted for another brief interview to get your reactions and opinions for what might work best in your practice?

---END--
